# Giant-magnetocaloric effect and phonon dynamics in (GdCe)CrO_3_

**DOI:** 10.1038/s41598-026-42301-9

**Published:** 2026-04-06

**Authors:** Ravi Kiran Dokala, Shaona Das, Subhash Thota

**Affiliations:** 1 Solid State Division, Department of Materials Science and Engineering, Uppsala University, Uppsala, 75237 Sweden; 2 Institute of Material Science, Technical University of Darmstadt, 64287 Darmstadt, Germany; 3 Department of Physics, Indian Institute of Technology Guwahati, Assam Guwahati, 781039 India

**Keywords:** Materials science, Physics

## Abstract

We investigate the effect of Ce^3+^ substitution on the magnetic ordering and phonon dynamics of the GdCrO_3_ orthorhombic perovskite. The Ce doped compound exhibits long-range canted antiferromagnetism with Néel transitions, *T*_N_ at ~ 173 K, accompanied by spin-flip, *T*_SF_ at ~ 10 K. Ce^3+^ incorporation drives a modification of the spin-flip transition from the ${\varGamma}_{4}\left({G}_{x},{A}_{y},{F}_{z}\right)$configuration to ${\varGamma}_{4}^{{\prime}}$ inducing a reorientation of the spin axis between the $\left(00\stackrel{-}{1}\right)$and (001) crystallographic planes. This spin reorientation is governed by Zeeman energy and produces pronounced field-induced irreversibility between FCC and FCW magnetization processes. The substituted compound Gd_0.9_Ce_0.1_CrO_3_ (GCCO) exhibits a remarkably large magnetic entropy change, $-\varDelta{S}_{M}$ ~ 45 –40 J/kg-K for ∆H = 90 − 70 kOe at 3 K among the highest reported for rare-earth orthochromites. The interplay of spin-only magnetocrystalline anisotropy from Cr^3+^ and spin–orbit–driven magnetic moments of Gd^3+^ and Ce^3+^ results in pronounced spin–phonon coupling, manifested through the A_1g_(6) vibrational mode. The observed temperature-dependent spectral evolution is consistent with behaviour reported in isostructural magnetic perovskites.

## Introduction

Heavier rare-earth (*R*) perovskites, particularly the *R*-chromates *R*CrO_3_ (R = Pr, Ce, Gd, Sm, etc.), exhibit highly complex magnetic ordering characterized by robust spin dynamics and strong exchange interactions among Cr^3+^–Cr^3+^, *R*^3+^–Cr^3+^ and *R*^3+^–*R*^3+^ sublattices^1,2^. Owing to these interactions, the chromate family demonstrates several intriguing physical phenomena such as spin-flip transitions, negative magnetization and compensation effects, multiferroicity, giant magnetocaloric response, and field-induced magnetic-phase transitions, which are of significant interest for the design and development of spin-valve and related spintronic devices^1,2^. Rare-earth orthochromites have been extensively investigated over the past several decades using a wide range of experimental techniques including heat capacity measurements, neutron diffraction, Mössbauer spectroscopy, magnetodielectric studies, and optical-absorption spectroscopy to probe their emergent ferroic and magnetic states^3–5^. These studies have demonstrated that the coexistence of local non-centrosymmetric structural distortions and multiple magnetic-ordering components (antiferromagnetic and ferromagnetic) plays a crucial role in governing the ferroelectric response in RCrO_3_. In addition, phenomena such as exchange bias, spin-reorientation transitions, negative magnetization and its reversal, and spin-glass-like behavior have also been widely reported in *R*CrO_3_-based systems^4–8^. Among these effects, magnetization reversal is particularly captivating due to its origin in the contrasting temperature dependencies of magnetic moments associated with distinct magnetic sublattices. In such compounds, the total magnetization switches sign from positive to negative below a characteristic temperature known as the compensation temperature (*T*_Comp_), a feature now regarded as a hallmark of *R*CrO_3_ systems^9^. Magnetization reversal, which relies on the presence of two competing magnetization components, renders these materials promising for thermally or magnetically driven switching technologies, including non-volatile magnetic memory storage, thermo-magnetic switches, high-speed read/write magnetic memory, and thermally assisted MRAM architectures^10^.

Most *R*CrO_3_ compounds, however, exhibit *T*_Comp_ significantly below room temperature, which limits their applicability in practical magneto-electronic devices. Consequently, enabling the tunability of *T*_Comp_ toward higher temperatures has become an important research objective. The internal molecular field generated by the transition-metal sublattice (Cr^3+^, Mn^3+^, etc.) plays a decisive role in driving magnetization reversal in *R*CrO_3_-type materials: rare-earth ions (e.g. Gd^3+^, Nd^3+^, Sm^3+^, and Ce^3+^) couple antiferromagnetically with the transition-metal ions, resulting in polarization and alignment of their magnetic moments opposite to the external field, ultimately yielding a net negative magnetization^11^. For most members of the *R*CrO_3_ series (e.g. PrCrO_3_, YbCrO_3_, HoCrO_3_), magnetization reversal is observed in the field-cooled warming (FCW) protocol. Remarkably, the end-member compound GdCrO_3_ displays negative magnetization only during the field-cooled cooling (FCC) cycle, highlighting the strong dependence of the magnetic response on the measurement history and protocol^12^.

In addition to their diverse magnetic characteristics, complex oxide chromates are widely recognized for exhibiting a substantial magnetocaloric effect (MCE), making them promising candidates for environmentally benign magnetic refrigeration technologies. Such systems offer an attractive alternative to conventional gas-compression refrigeration, which continues to dominate global markets despite its environmental hazards^13^. The magnetic interactions driving exotic phase transitions within the constituent magnetic sublattices enable these materials to operate as refrigerants across a broad temperature range. Depending on the transition temperatures involved, chromates hold strong potential for hydrogen and helium liquefaction, thereby competing effectively with well-established intermetallic MCE systems such as GdNi_2_ and La(Fe, Si)_13_^14,15^. Previous studies have reported a giant MCE in polycrystalline GdCrO_3_ with $-\varDelta{S}_{\mathrm{M}}$ ~ 36.97 JKg^−1^K^−1^, the magnitude of $-\varDelta{S}_{\mathrm{M}}$can be further enhanced by tailoring selective magnetic interactions that reinforce the overall magnetic moment^4^.

Among chromates, GdCrO_3_ and CeCrO_3_ are prototypical antiferromagnetic systems well known for displaying magnetization-reversal behavior^3,4^. GdCrO_3_ is a G-type AFM compound with *T*_N_ ~ 169 K and a compensation temperature *T*_Comp_ ~ 132 K; a spin-flip transition occurs at *T*_SF_ ~ 18 K at fields as low as 100 Oe. A giant MCE has also been reported at low temperatures with $-\varDelta{S}_{\mathrm{M}}$ ~ 36.97 JKg^−1^K^−1^ at 70 kOe. CeCrO_3_ also exhibits a G-type AFM spin configuration, though its spin-flip transition takes place at higher magnetic fields and ultimately compensates the magnetization, driving it into the positive regime. According to the report by Cao et al., CeCrO_3_ displays *T*_N_ ~ 230 K, *T*_Comp_ ~ 100 K, and *T*_SF_ ~ 36 K at 1.2 kOe^3^.

In the present work, we investigate the polycrystalline chromate perovskite system GCCO by examining its structural, electronic, and magnetic properties. X-ray diffraction and Raman spectroscopy provide insights into the internal atomic structure and its response to vibrational excitations. The GCCO system demonstrates magnetization reversal, a field-tunable spin-flip transition, and exchange bias—indicative of multiple coexisting magnetic configurations. Furthermore, GCCO exhibits a giant magnetocaloric effect, attributed primarily to local structural distortions introduced by substituting trivalent Ce^3+^ at the relatively smaller Gd^3+^ sites.

## Methods

Polycrystalline GCCO was synthesized by a standard solid-state reaction route. Stoichiometric amounts of Gd_2_O_3_ (99.9%), CeO_2_ (99.95%), and Cr_2_O_3_ (99.99%) were thoroughly ground in an agate mortar for 5 h and calcined at 1000 °C for 24 h in air to ensure homogeneity. The calcined powder was reground for 2 h, pelletized, and sintered at 1200 °C for 24 h in a Nabertherm tubular furnace, followed by natural cooling to room temperature. Phase purity was confirmed using room-temperature X-ray diffraction (Rigaku TTRAX III, Cu-Kα_1_, λ = 1.54056 Å, step size 0.02°). X-ray photoelectron spectroscopy (XPS) was carried out using a PHI 5000 VersaProbe III system with an Al Kα source to determine the core-level charge states. DC magnetization measurements were performed using a Quantum Design PPMS (Dyna-Cool model). Magnetization was recorded as a function of temperature (3–300 K) and magnetic field (50 Oe–20 kOe) under zero-field cooled warming (ZFCW), field-cooled cooling (FCC), and field-cooled warming (FCW) protocols. Isothermal M–H loops were measured between ± 90 kOe at selected temperatures, and time-dependent magnetization was recorded under the FCC protocol. Room-temperature Raman spectra (100–800 cm^− 1^) and low-wavenumber scans (100–200 cm^− 1^) were obtained using a Horiba Jobin Yvon LabRam HR spectrometer with a 20 mW He–Ne laser. Temperature-dependent Raman measurements down to 80 K were conducted using a THMS600 module.

## Results and discussion

**Fig. 1:**
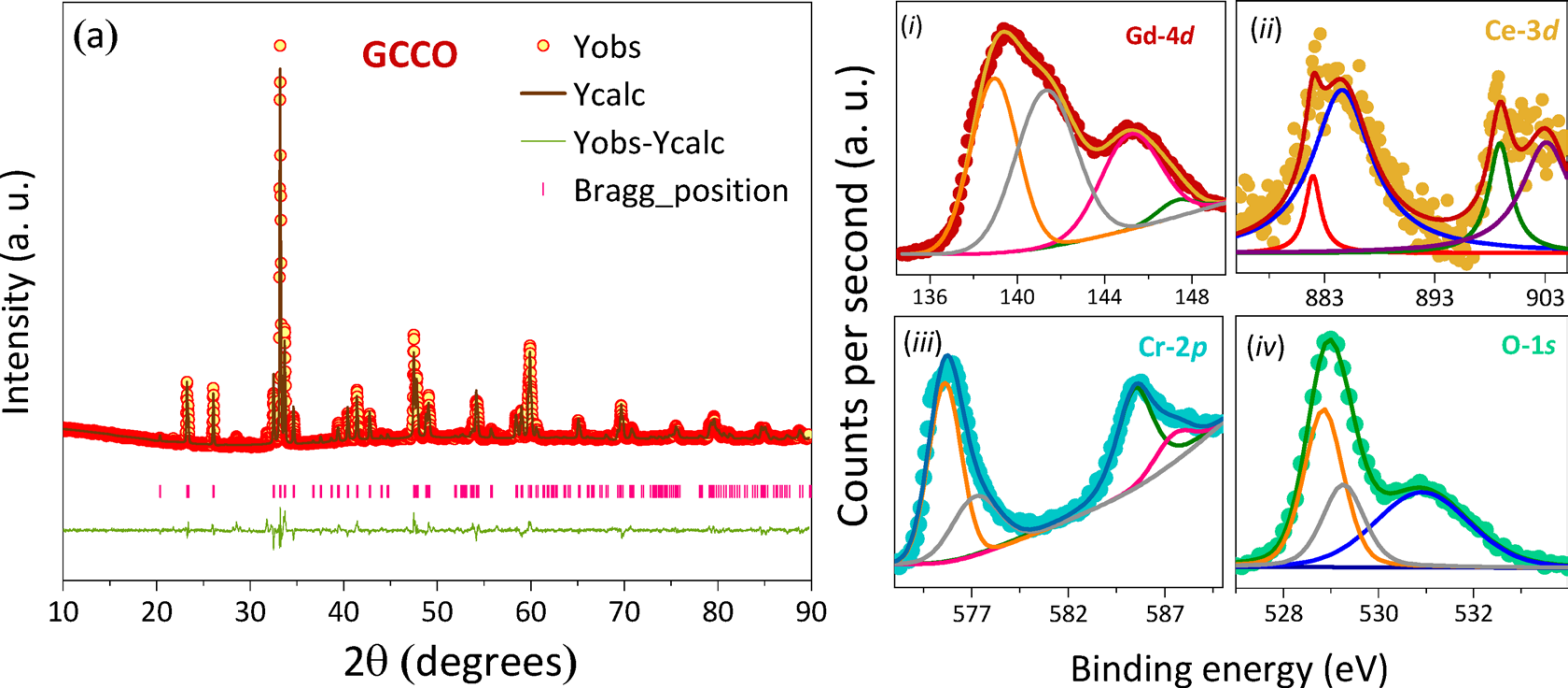
(**a**) Rietveld refined X-ray diffraction patterns of GCCO showing single phase *Pbnm* orthorhombic perovskite structure. X-ray photoelectron spectroscopy of GCCO (*i*) $\mathrm{G}\mathrm{d}-4d$, (*ii*) $\mathrm{C}\mathrm{e}-4d$, (*iii*) $\mathrm{C}\mathrm{r}-2p$ (*iv*) $\mathrm{O}-1s$. Scattered symbols represent the original data and solid lines are the fitted curves.

GdCrO_3_ and CeCrO_3_ typically crystallize in a slightly distorted orthorhombic perovskite structure with space group *Pbnm*^16–19^. In GCCO, the larger Ce^3+^ ions substitute at the *A*-site (12-fold coordination) without altering the *B*-site Cr^3+^ environment (6-fold coordination). Figure 1 shows the XRD patterns of the GCCO samples, confirming single-phase formation without secondary impurities. Rietveld refinement was performed using FULLPROF^20^, and all Bragg reflections were indexed to the *Pbnm* (No. 62) structure. The obtained goodness-of-fit values (χ^2^ = 2.42 and 2.63) indicate reliable refinement. The *A*-site occupancy of Gd/Ce was refined and found to be consistent with the nominal stoichiometry. Ce substitution leads to slight increases in the lattice parameters *a* and *b*, consistent with the larger ionic radius of Ce^3+^ (1.143 Å) compared to Gd^3+^ (1.053 Å; 8-coordination). Consequently, the unit-cell volume increases by ~ 0.072%, and the *c* parameter also shows a small positive shift. In order to understand the internal crystallographic behaviour, we tabulated all the structural parameters refined and calculated along with the comparative study of the pristine compounds from the previously reported values in Table 1. These changes can be explained with the help of difference of average radius of *A-*site cation: ${r}_{\mathrm{a}\mathrm{v}\mathrm{g}}=\sqrt{\left[\left(0.9\right)\times{R}_{{\mathrm{G}\mathrm{d}}^{3+}}^{2}\right]+(0.1\times{R}_{{\mathrm{C}\mathrm{e}}^{3+}}^{2})}$ for GCCO sample which increases to 1.063 Å compared to 1.053 Å for pristine compound with ${R}_{{\mathrm{G}\mathrm{d}}^{3+}}$ = 1.053 Å having VIII coordination number and that of ${R}_{{\mathrm{C}\mathrm{e}}^{3+}}$ = 1.143 Å having the same coordination number^21^. The substitution of larger cation impacts the crystal symmetry by introducing significant distortion of the unit-cell which is popularly known as the factor of tolerance (*t*), given in the following Eq. (1).

1 $$t=\frac{\left\{\right[(1-x)\times{R}_{{\mathrm{G}\mathrm{d}}^{3+}}]+(x\times{R}_{{\mathrm{C}\mathrm{e}}^{3+}})+{R}_{{\mathrm{O}}^{2-}}\}}{\sqrt{{({R}_{\mathrm{C}\mathrm{r}}^{3+}+R}_{\mathrm{O}}^{2-})}}$$

**Table 1:** The refined crystallographic parameters obtained from XRD of GCCO. The parameters, *a*,* b*,* c*,* and V* are lattice constants and lattice volume respectively. θ_1_ and θ_2_ are the Cr-O_(1)_-Cr and Cr-O_(2)_-Cr bond angles. θ and ϕ are the in-phase and out-of-phase tilt angles *w.r.t.* 110] and [001] respectively. ∆, octahedral distortion, *t*, tolerance factor, *r*_avg_, average radius of the *R*^3+^ cation.

Parameter		Atomic positions		Bond lengths		Bond angles		Other parameters		
*a* *b* *c* V	5.31946 5.50727 7.60545 222.807	Gd/Ce (4c)	0.99365 0.05841 0.25000 1.000	Gd/Ce-O_(1)_ (*two*) Gd/Ce-O_(2)_ (*six*) Cr-O_(1)_ (*two*) Cr-O_(2)_ (*four*)	2.3256(0) 2.3779(0) 2.6429(0) (*two*) 2.5845(0) (*two*) 2.2850(0) (*two*) 1.9464(0) (*two*) 1.9239(0) (*two*) 2.0845(0) (*two*)	θ_1_ θ_2_	155.3 145.5	θ [110] ϕ [001]θ (*from lattice parameters*)ϕ (*from lattice parameters*) r_avg_ *t* ∆ (⨯10^− 4^) s ᶲ	12.36 14.92 8.45 15.01 1.062 0.8663 1.2800 0.03469 17.1748	
		Cr (4b)	0.00000 0.50000 0.00000 1.000							
		O1 (4c)	0.07624 0.48276 0.25000 0.900							
		O2 (8b)	0.68067 0.29345 0.05355 1.599							

Accordingly, the octahedral distortion (∆) can be expressed as below in Eq. (2).

2 $$\Delta = \left( {\frac{1}{N}} \right)\mathop \sum \limits_{{n = 1,N}} ~\{ (d_{n} - \{ d)){ \nless }d> \} ^{2}$$

Here, the apical Cr-O_(1)_-Cr bond angle (*θ*_1_), basal Cr-O_(2)_-Cr bond angle (*θ*_2_) and the tilt angles θ and ϕ inside the CrO_6_ octahedra along the pseudo-cubic axes [110] and [001] calculated from the bond angles that exhibit visible deviations with the *A*-site cation substitution than that of the average bond lengths between the Cr and the O anions (changes ~ 0.54%) directing towards the bond rigidity expected in octahedral symmetry surrounding the trivalent Cr^22^. Also, we have calculated the tilt angles from the experimentally obtained lattice parameters, ${\uptheta}={cos}^{-1}\frac{\surd2a}{b}$, ${\upphi}={cos}^{-1}\frac{a}{b}$ and the magnitudes match well with the previously reported values of similar systems^23,24^. Along with the tilting of the CrO_6_ octahedra, due to the mismatch between the A -site Gd/Ce and B-site Cr cations, an impulsive reduction in the strain parameter *s* with an increment of ~ 2.2% is observedas the average radius of the *A*-site cation comprises the Wyckoff position eccentricities of Gd/Ce (4c) and the Cr (4b) sites.

X-ray photoelectron spectroscopy (XPS) was employed to examine the electronic structure, oxidation states, and surface composition of the synthesized GCCO perovskites. Figure 1 (*i–iv*) presents the room-temperature high-resolution spectra, where the scattered points correspond to experimental data and the solid curves represent Lorentzian–Gaussian fitting results. The spectra were collected with a binding-energy resolution of 0.1 eV, and the background was corrected using the Tougaard method. Peak fitting was performed through a nonlinear least-squares procedure, with the binding-energy scale calibrated to the C 1 s peak at 285 eV. Core-level spectra of Gd-4*d*, Ce-3*d*, Cr-2*p*, and O-1*s* were analyzed individually, and all obtained binding energies matched well with standard NIST values^25^. The Gd-4*d* spectrum shows two clear spin–orbit doublets corresponding to the 4d_5/2_ and 4d_3/2_ states. Peaks at 139.1, 141.4, 145.3, and 147.4 eV agree with literature values for Gd^3+^^26–28^. Additional lower-energy components (138.4 and 140.8 eV) also arise from the 4d_5/2_ level. The goodness-of-fit (χ^2^ ≈ 2.7) confirms reliable peak deconvolution, and the absence of satellite structures indicates that Gd is stabilized in the trivalent state. Figure 1(*ii*) displays the Ce-3*d* core-level spectrum, containing four major peaks at 882.1, 884.7, 899.3, and 903.5 eV. These features originate from the 3d_5/2_ and 3d_3/2_ components and correspond to Ce^3+^ states^30^. Although Ce frequently exhibits Ce^3+^/Ce^4+^ mixed valence in oxides, no signature of Ce^4+^ was observed in GCCO, confirming the chemical homogeneity and phase purity. Overall, the XPS results validate that all constituent elements retain their expected oxidation states, with no evidence of extrinsic phases or additional oxidized species.

**Fig. 2:**
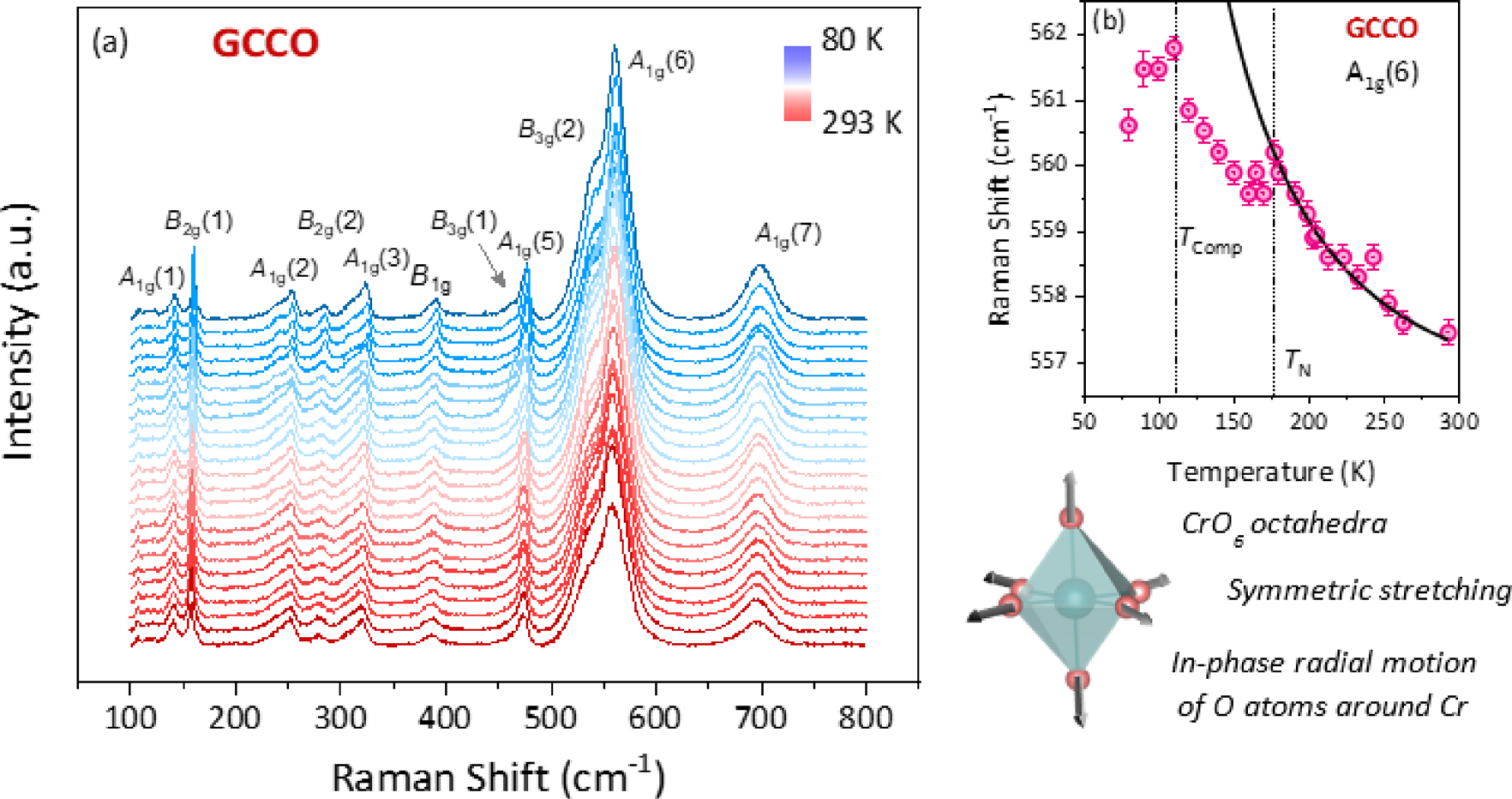
(**a**) Raman spectra with all the existing modes indexed for GCCO from 80 K to 293 K. Self-explanatory schematic of symmetric stretching mode, along with temperature dependence of A_1g_(6) mode.

Raman spectroscopy is a sensitive probe of lattice vibrations, octahedral tilts, and local distortions in orthorhombic perovskite oxides, offering insight into cation displacements and bond-strength variations that influence their physical properties. It is particularly effective in detecting subtle symmetry changes arising from cation-size mismatch, such as that between Gd^3+^ (1.053 Å) and Ce^3+^ in GCCO. In rare-earth chromites, Raman-active phonons reflect the stability of the CrO_6_ octahedra and their coupling to the *A*-site environment. Figure 2a shows the temperature-dependent Raman spectra of GCCO (80–293 K, 100–800 cm^− 1^). Based on the orthorhombic *Pbnm* structure with Glazer tilt system a^−^b^+^a^−^, group theory predicts 60 modes, of which 24 are Raman active (7A_1g_ + 5B_1g_ + 7B_2g_ + 5B_3g_)^21–23^. These correspond to octahedral stretching, bending, rotational modes, and *A*-site cation motions. As Cr^3+^ (3*d*^3^) is Jahn–Teller inactive, distortions mainly arise from octahedral tilts and *A*-site effects^22^. Eleven Raman modes are observed experimentally, showing temperature-dependent red- and blue-shifts indicative of anharmonic phonon interactions.

The low-frequency phonons (< 200 cm^− 1^) in orthorhombic chromite perovskites are primarily governed by rare-earth *A*-site cation vibrations, whose frequencies depend on the reduced mass (µ) according to ω = √(k/µ), where k is the effective force constant. In GCCO, these low-energy modes are assigned as A_1g_ (1) and B_2g_ (1). Above 200 cm^− 1^, the phonon modes arise from vibrations involving Gd/Ce and oxygen atoms as well as bending and stretching motions of the Cr–O bonds. Among these, the A_1g_ (1) and B_2g_ (1) doublets originate from collective octahedral rotations around the y-axis characteristic of the a^−^b^+^a^−^ tilt system. At higher frequencies, the mode at ~ 561 cm^− 1^ (80 K) corresponds to CrO_6_ octahedral bending, while the intense 698 cm^− 1^ peak reflects antisymmetric octahedral stretching that is highly sensitive to Cr–O bond-length and bond-angle variations. The stability of these modes, along with their moderate and systematic shifts with temperature, confirms the absence of structural phase transitions across the measured range and supports the robustness of the orthorhombic phase.

Temperature-dependent Raman analysis reveals systematic softening and hardening trends consistent with anharmonic phonon behavior. For GCCO, the A_1g_(6) mode shows a clear temperature-dependent shift toward lower wavenumbers upon warming, directly linking changes in (Gd/Ce)/Cr–O bond lengths and O–Cr–O bond angles to the vibrational response. Figure 2b illustrates the fitted A_1g_(6) mode near *T*_N_, where the peak positions were extracted using Lorentzian fitting. To examine the interplay between lattice vibrations and magnetism, the Raman line-shape parameters were analyzed across the magnetic ordering temperature. A subtle but distinct kink at *T*_N_ indicates weak yet measurable spin–phonon coupling (SPC). Similar weak anomalies have been reported in other *R*CrO_3_ systems, often attributed to the interplay between rare-earth magnetism, Cr–O–Cr superexchange, and structural distortions.

The temperature-dependence of the phonon-mode with frequency ω follows the conventional relation^29^:

3 $$\omega\left(T\right)={\omega}_{0}\left(T\right)+\varDelta{\omega}_{lat}\left(T\right)+\varDelta{\omega}_{sp-lat}\left(T\right)+\varDelta{\omega}_{el-ph}\left(T\right)+\varDelta{\omega}_{anh}\left(T\right)$$

Here, ω₀ is the harmonic frequency at 0 K, $\varDelta{\omega}_{latt}$ the quasi-harmonic lattice-volume contribution, $\varDelta{\omega}_{sp-lat}$ the spin–lattice coupling term, $\varDelta{\omega}_{el-ph}$the electron–phonon component (negligible for insulating chromites), and $\varDelta{\omega}_{anh}$ the intrinsic anharmonic contribution. For GCCO, where electron-phonon coupling is insignificant and lattice effects are modest, the frequency evolution reduces to:

4 $$\omega\left(T\right)=\varDelta{\omega}_{sp-lat}\left(T\right)+\varDelta{\omega}_{anh}\left(T\right)$$

The dominant mechanism above *T*_N_ is the anharmonic phonon-phonon interaction, which leads to frequency hardening as temperature decreases. This contribution is modelled using the standard three-phonon and four-phonon decay processes^24^:

5 $$~~\omega _{{anh}} ~\left( T \right) = \omega _{0} + A\left[ {1 + \frac{2}{{e^{{\frac{{~\hbar \omega _{0} }}{{2k_{B} T}}}} - 1}}} \right] + B\left[ {1 + \frac{3}{{e^{{\frac{{~\hbar \omega _{0} }}{{3k_{B} T}}}} - 1}} + ~\frac{3}{{\left( {e^{{\frac{{~\hbar \omega _{0} }}{{3k_{B} T}}}} - 1} \right)^{2} }}} \right]$$

where, the parameters A and B were obtained by fitting temperatures above *T*_N_, and the resulting curves were extrapolated below *T*_N_ to highlight deviations clearly attributable to magnetic ordering. As shown in Fig. 2b, the A_1g_ (6) mode of GCCO exhibits an anomalous softening immediately below *T*_N_, followed by hardening at lower temperatures. This behavior reflects the competition between exchange-striction effects (which soften the mode as spins begin to order) and stabilization of the long-range antiferromagnetic state (which increases restoring forces at lower T). The observed ~ 4 cm^− 1^ total shift of A_1g_ (6) mode from $\sim$557 cm^−1^ at 300 K to $\sim$561 cm^− 1^ at 80 K further confirms this interplay.

To quantify SPC strength, the Granado model is invoked, where phonon renormalization scales with the nearest-neighbour spin correlation^29^:

6 $$\varDelta{\omega}_{sp-lat}\propto<{S}_{i}.{S}_{j}>$$

In the molecular-field approximation:

7a $$\Delta ~\omega _{{sp - lat}} \approx S^{2} [1 - (T/T_{N} )\gamma ]$$

7b $$\approx\lambda{\left[\frac{M\left(T\right)}{{M}_{sat}}\right]}^{2}$$

For GCCO, fitting these expressions produced *λ* values fluctuating between 0.3 and 0.7 cm^− 1^, reflecting the non-monotonic phonon behavior arising from the abrupt softening near *T*_N_ and subsequent hardening. Such complexity is typical of systems where octahedral tilts and A-site disorder influence local exchange pathways.

Further insight into SPC comes from structural parameters: the CrO_6_ octahedral distortion Δ, extracted from Rietveld refinement, is 1.28 × 10^− 4^ for GCCO, indicating weak static distortion. Even this small Δ can significantly affect the Cr–O–Cr exchange integral, which is highly sensitive to bond-angle variations (following Goodenough–Kanamori rules). The rare-earth magnetic moments also play a key role: Gd^3+^ (µ_eff_ = 7.94 µ_B_, *L* = 0) contributes primarily spin-only magnetism, whereas Ce^3+^ (µ_eff_ = 2.54 µ_B_, L = 3) introduces moderate spin–orbit coupling. Their combined presence in GCCO results in a weaker net SPC, consistent with the modest Raman anomalies observed. Overall, the Raman analysis confirms that both octahedral distortions and rare-earth magnetism influence spin–phonon interactions in GCCO, with the A_1g_(6) mode serving as the clearest indicator of this coupling. Besides, a notable difference between GCCO and undoped GdCrO_3_ emerges in the 500–600 cm^− 1^ region, where the CrO_6_ symmetric stretching vibrations occur. In GdCrO_3_, the high-frequency A_1g_(7) stretching mode is present but comparatively weak and does not dominate the spectrum^31,32^. In contrast, in GCCO the corresponding A_1g_(6) mode becomes the strongest and most pronounced Raman feature, indicating a substantial enhancement of the symmetric CrO_6_ breathing vibration. The markedly higher spectral intensity suggests that Ce^3+^ substitution increases the polarizability of the in-phase radial oxygen motions and modifies the local Cr–O bond covalency. This observation aligns with structural refinements showing altered Cr–O–Cr bond angles and increased octahedral distortions, demonstrating that A-site chemical substitution not only affects the phonon energies but also amplifies the vibrational strength of the CrO_6_ stretching mode. Such enhancement supports the conclusion that Ce doping strengthens the coupling between lattice dynamics and magnetic interactions which we discuss further in the following sections.

**Fig. 3:**
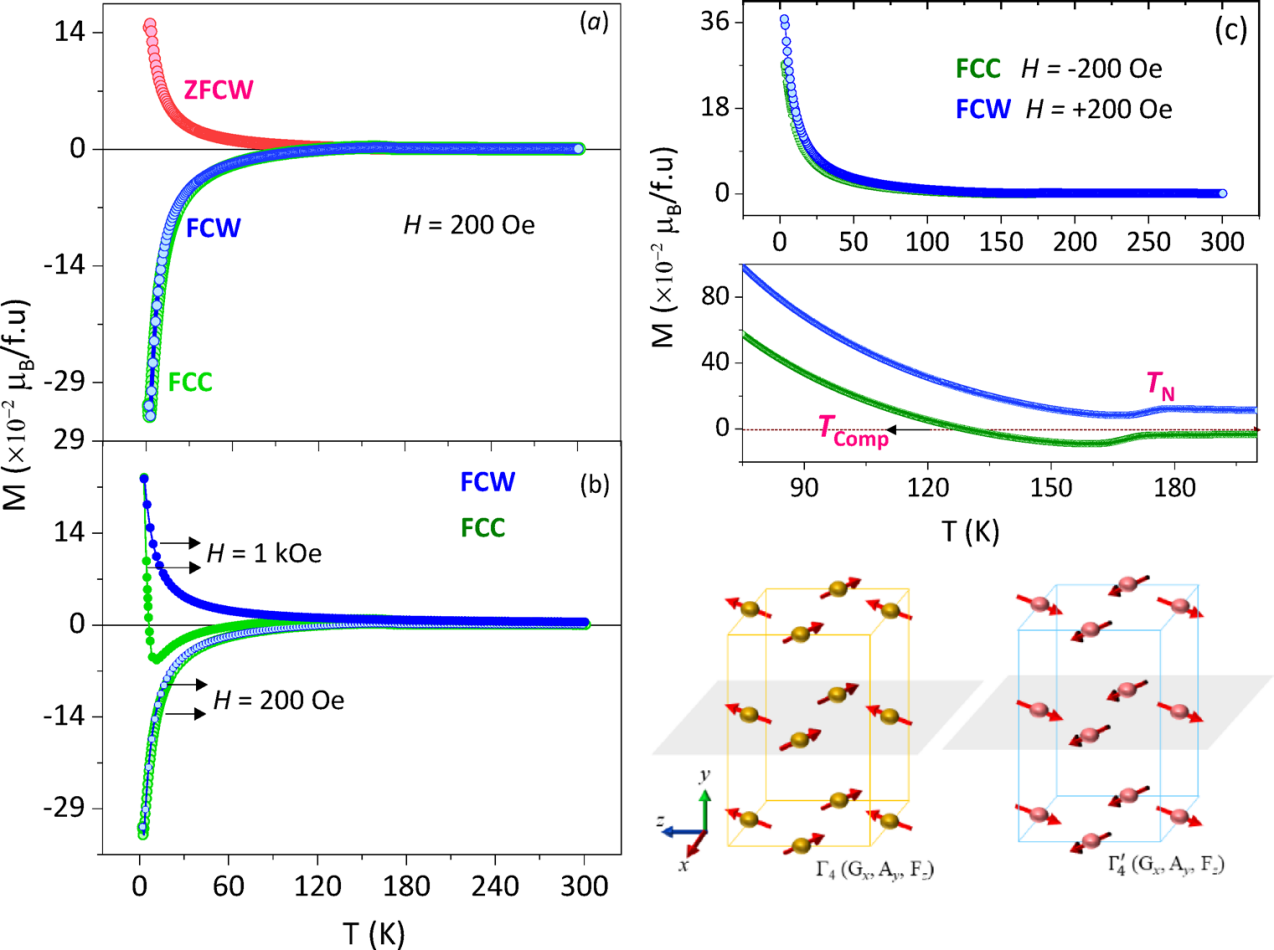
(**a**) Temperature dependent magnetization of GCCO under (a) ZFCW, FCC, and FCW protocols. (**b**) FCC and FCW at field 1kOe and 200 Oe. Irreversible Spin-flip transition observed during the FCC at 1 kOe. (**c**) FCC under the applied field *H* = −200 Oe and FCW under the field *H* = + 200 Oe. Zoomed view given below to show the *T*_Comp_ observed only under the FCW condition.

Figure 3a exhibits the temperature dependent magnetization under ZFCW, FCC and FCW protocols. Under *H*_DC_ = 100 Oe, the three protocols exhibit the long-range magnetic ordering suggesting the canted anti-ferromagnetism with Néel temperature at ${T}_{\mathrm{N}}$ ~ 173.4 K which is confirmed by the $\frac{d\left(\chi\mathrm{T}\right)}{d\mathrm{T}}$ vs. T with Curie-Weiss linear fit that ventures into the negative temperature scale and meets the *X*-axis at Curie-Weiss temperature, ${\varTheta}_{\mathrm{D}}$ ~ −28.6 K by extrapolating the linear fit at the high temperature paramagnetic behaviour^30^. Long-range magnetic ordering lead by the Cr^3+^ sublattice configured in the G-type CAFM structure identified with the Bertaut’s notation ${\varGamma}_{4}\left({G}_{x},{A}_{y},{F}_{z}\right)$ as shown in Fig. 3^16^. The negative magnetization popped up during the FCC condition meets the compensation point at ${T}_{\mathrm{C}\mathrm{o}\mathrm{m}\mathrm{p}}$ ~ 121 K. Beyond the ${T}_{\mathrm{C}\mathrm{o}\mathrm{m}\mathrm{p}}$, the Gd^3+^ and Ce^3+^ contribution increases which is evident through the increase in negative magnetization and attains a maximum value of $34\times{10}^{-2}\frac{{\mu}_{\mathrm{B}}}{f.u.}$ under *H*_DC_ = 100 Oe. From Fig. 3b, under an applied magnetic field of 1 kOe, the magnetization in negative scale suddenly switches to the positive under FCC condition which can be understood as spin-flip transition which is well-known for GdCrO_3_ polycrystalline system^4^. Beyond the Spin-flip transition temperature, ${T}_{\mathrm{S}\mathrm{F}}$ ~ 10 K the magnetic sublattice gains the Zeeman energy which is helpful for flipping the spins from ${\varGamma}_{4}\left({G}_{x},{A}_{y},{F}_{z}\right)$ to ${\varGamma}_{4}^{{\prime}}\left({G}_{x},{A}_{y},{F}_{z}\right)$ from $\left(00\stackrel{-}{1}\right)$ to $\left(001\right)$ where, the *c*-axis is the easy axis^3^. The parameters and their influence on Zeeman energy of the system is given by the following Eq. (8):

8 $${E}_{zeeman}=-{\mu}_{0}{M}_{\mathrm{N}\mathrm{e}\mathrm{t}}{H}_{\mathrm{E}\mathrm{x}\mathrm{t}}\mathrm{C}\mathrm{o}\mathrm{s}{\uptheta}$$

where ${\uptheta}$ is the angle between the ${M}_{\mathrm{N}\mathrm{e}\mathrm{t}}$, net magnetization and ${H}_{\mathrm{E}\mathrm{x}\mathrm{t}}$, externally applied magnetic field. Net moments with ${\uptheta}=\pi$ contains large Zeeman energy and they are easy to be flipped, on the other hand the spins with ${\uptheta}<\pi$ will be flipped by applying more external magnetic field. The spins flipped increase the magnetization in the positive scale by crossing the magnetization compensation. During the FCW condition, the curve cannot reverse into the ${\varGamma}_{4}\left({G}_{x},{A}_{y},{F}_{z}\right)$ configuration but continue in the ${\varGamma}_{4}^{{\prime}}\left({G}_{x},{A}_{y},{F}_{z}\right)$ condition and attains PM configuration after the ${T}_{\mathrm{N}}$. Spin-flip phase transition occurred in this system is irreversible similar to the GdCrO_3_ and CeCrO_3_. From Fig. 3c, the magnetization measurements performed under FCC at ${H}_{\mathrm{D}\mathrm{C}}$ ~ −200 Oe and FCW at ${H}_{\mathrm{D}\mathrm{C}}$ ~ +200 Oe in order to understand the behaviour of magnetic ordering through the applied field polarity. While field cooled cooling protocol the magnetic ordering is exactly opposite to the magnetic ordering given in the Fig. 3a, signifying the parallel alignment of the Cr^3+^ sublattice to the applied field and anti-parallel alignment of the Gd^3+^ and Ce^3+^ moments to the local-field applied by the Cr^3+^ sublattice. At the frozen state of the magnetic-moments at 3 K, the field is changed from − 200 Oe to + 200 Oe and the magnetization is recorded while warming till temperatures above *T*_N_. An intriguing trend has been evident through positive magnetization along with an offset from the FCC magnetization shown all through the FCW protocol above the *T*_N_ even into the PM region. This very behaviour shows the characteristic pinned AFM between the Cr^3+^ and Gd^3+^/Ce^3+^ magnetic sublattices irrespective of the applied magnetic field polarity.

**Fig. 4:**
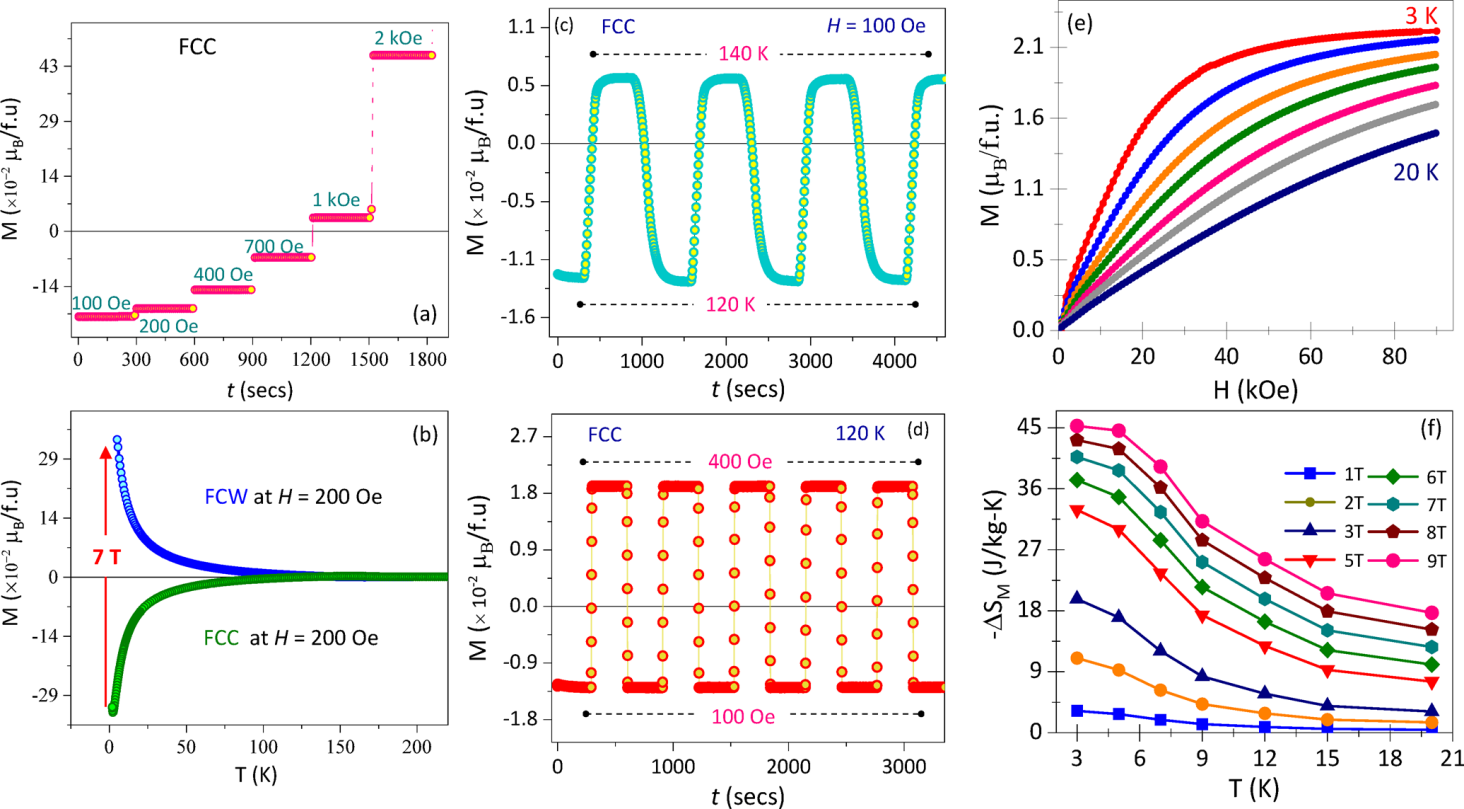
(**a**) Magnetization versus time measurements at different magnetic fields 100 Oe, 200 Oe, 400 Oe, 500 Oe, 700 Oe, 1 kOe, 1.3 kOe, 2 kOe representing the gradual increase in the no. of spins that flip *w.r.t.* applied field. (**b**) Temperature dependent magnetization under the FCC condition by applying external field of *H* = 200 Oe. Raise the magnetic field to 70 kOe at 5 K. Followed by decreasing the magnetic field again to 200 Oe and measurement was recorded under FCW condition. Time stamp measurements of (**c**) temperature dependent magnetization switching of GCCO between 120 K and 140 K under FCC condition at applied field, *H* = 100 Oe, (**d**) field dependent magnetization switching between 400 Oe and 100 Oe under FCC condition at 120 K. (**e**) Magnetic field dependence of magnetization of GCCO at different temperatures from 3 K to 20 K under ZFC. (**f**) Temperature dependence of isothermal entropy change, −∆*S*_M_.

In order to understand the field dependent magnetic sublattice behaviour and the stability of the spins that gets flipped, time stamp magnetization measurements at 3 K at different magnetic fields, *H*_DC_ = 100 Oe, 200 Oe, 400 Oe, 500 Oe, 700 Oe, 1 kOe, 1.3 kOe, and 2 kOe under FCC condition as shown in the Fig. 4a. The overall magnetization M(T) undergoing the second transition at the low temperatures associated with spin-flip transition triggered by the critical field, *H*_C_ = 200 Oe but not enough to fully establish a spin-flipped magnetic sublattice. The gradual spin-flip transition is tailored by gradually changing the external magnetic fields and witness a completely compensating sublattice’s spin-flip configuration from 1 kOe and above magnetic fields. The number of spins flipped depends on the amount of magnetic field applied which is clearly seen from Eq. 7. The sample has been cooled under an applied filed of 100 Oe to 3 K where the negative magnetization persists as the Zeeman energy is not enough to flip the sublattice into ${\varGamma}_{4}^{{\prime}}$ configuration. Magnetization has been measured for 300 s followed by measuring the magnetization at 200 Oe for 300 s and increasing the field to 400 Oe, 700 Oe, 1 kOe and 2 kOe. The number of spins flipped and magnetization shooting up to the positive scale explicitly observed through the time stamps confirming their stability and shows the robust dependency of the Zeeman energy on the externally applied magnetic field. The path-independent nature of the spin flip transition has been observed through the cooling and heating under different magnetic fields, CHUF protocol as shown in the Fig. 4b^33^. The magnetization recorded under the FCC condition by applying a magnetic field of *H*_DC_ = 200 Oe. This reflects the absence of the spin-flip transition. At 3 K the magnetic field has been raised to 70 kOe, and the FCW curve has been recorded while warming. The magnetization curve during the warming cycle reflects the completely flipped spins containing the ${\varGamma}_{4}^{{\prime}}$ configuration.

The stability and reversibility of magnetization polarity in GCCO were examined through time-dependent magnetization measurements. Figure 4c shows the temperature-controlled switching under an applied field of 100 Oe in the FCC protocol. The sample was first cooled to 120 K, below *T*_Comp_, where negative magnetization appears, and the magnetization was recorded for 300 s. The temperature was then raised to 140 K (above *T*_Comp_) and measured for another 300 s. This cycle following 120 K → 140 K → 120 K → 140 K was repeated multiple times. Across each temperature transition, the magnetization consistently switched between negative and positive values without decay, maintaining stability for more than 4000 s. Figure 4d presents field-controlled switching at 120 K under FCC conditions. The sample was held at 100 Oe for 300 s, followed by an increase to 400 Oe for 300 s, and then returned to 100 Oe all at constant temperature. This sequence was repeated five times. The magnetization reversibly follows the applied field, demonstrating stable spin alignment and switching behaviour. Since the system remains above *T*_SF_, it avoids entering the spin-flipped ${\varGamma}_{4}^{{\prime}}$ configuration, enabling consistent switching. These results confirm reliable, repeatable magnetization polarity control, highlighting GCCO’s potential for magnetic switching device applications^27,34,35^.

In order to investigate the influence of the *A*-site doping at on the magnetocaloric effect (MCE), we executed the first quadrant ZFCW isotherm hysteresis curves at different temperatures and within the field range from 0 Oe to + 90 kOe. The experimental data is plotted in Fig. 4e for specific temperatures within 3 K to 20 K which includes the *T*_SR_ region. The MCE shows a direct proportional relation with the temperature derivative of magnetization (∂M/∂T)^36^:

9 $$\Delta S_{M} = ~\mathop \sum \limits_{i} \frac{{M_{{(T_{{i + 1}} ,~H) - }} M_{{(T_{i} ,~H)}} }}{{T_{{i + 1 - }} T_{i} }}\Delta H$$

10 $$~~\Delta S_{M} ~ = \mathop \smallint \limits_{0}^{H} \left( {\frac{{\partial M\left( {T,H} \right)}}{{\partial T}}} \right)dH$$

with $\varDelta{S}_{M}$as the isothermal magnetic entropy change. From the above equation, it can be easily interpreted that higher the value of (∂M/∂T), larger the MCE value.

On integrating the (∂M/∂T) with respect to chosen field values, the $\varDelta{S}_{M}$ curves are plotted in Fig. 4f. The resulting Δ${S}_{M}$(T) curves, exhibit smooth broad maxima rather than sharp peaks. With increasing field change (ΔH), the amplitude of these maxima increases monotonically, indicating field-enhanced spin alignment. A noticeable rise in Δ${S}_{M}$ occurs near T_SR_ ≈ 3 K, consistent with the field-induced ordering of the Gd^3+^ moments. This behavior is characteristic of orthorhombic rare-earth chromites, where the Cr^3+^ sublattice orders at high temperature and the rare-earth sublattice provides a low-temperature contribution to MCE.

We tabulated the comparative $\varDelta{S}_{M}$ values with the previously reported similar systems in Table 2. Gd has already attained great attention for its higher MCE value as well as the potential candidate for the magnetocaloric refrigeration with the $\varDelta{S}_{M}$ value of 10.2 J/kg-K^37^. The pristine GdCrO_3_ polycrystalline material holds $\varDelta{S}_{M}$ value as large as 38.7 J/kg-K under ∆H = 70 kOe at 5 K^38^ and hence the highest till date among the Gd systems. In our sample we attained a value of 40.70 J/kg-K under ∆H = 70 kOe at 3 K and even higher (45.27 J/kg-K) with a cumulative measurement error of value ~ 0.011 at ∆H = 90 kOe which is the new maximum among the rare earth orthochromite and even among the Gd based system in other crystal systems as well. Here, we can infer those numerous factors can control the MCE value for this A-site doped distorted system. One of these factors can be: this sample shows higher magnetization than the similar compounds with small coercive field leading to preserving more energy during the thermal process. Other than this, other elements possess higher magnetic moment (Dy ~ 10.63 *µ*_B_)^12^ but as the $\varDelta{S}_{M}$ depends on the thermal derivative, the slope for this particular sample is higher resulting to enhancement in $\varDelta{S}_{M}$. The magnetic spin reorientation near *T*_SR_ (~ 3 K) corresponds to the ordering of the Gd^3+^ spins due to the Gd^3+^-Gd^3+^ interaction greatly influences the $\varDelta{S}_{M}$ value. In addition, the comparative analysis summarized in Table 3 clearly highlights the multifaceted impact of Ce^3+^ substitution on the structural, vibrational responses and consequently on magnetic response of GdCrO_3_. Even the small replacement of Gd^3+^ with the larger Ce^3+^ ion leads to measurable lattice relaxation, reflected by reduced lattice constants, a slight contraction of the unit-cell volume, and systematic modifications in the Cr–O–Cr geometry. Specifically, Ce doping decreases the out-of-plane Cr–O–Cr angle from 146.5° to 145.5° while substantially increasing the in-plane angle from 148.9° to 155.3°, indicating a redistribution of octahedral tilts and modified CrO_6_ connectivity. These geometric changes lower the tolerance factor and subtly increase the orthorhombic strain, indicating a more distorted but structurally responsive octahedral framework. Raman spectroscopy captures these distortions most clearly through the CrO_6_ symmetric stretching mode shifts from ~ 560 cm^− 1^ in GdCrO_3_ to ~ 557.5 cm^− 1^ in GCCO and, more importantly, becomes the highest-intensity phonon mode in the doped compound, in stark contrast to the weak corresponding mode in pristine GdCrO_3_. The dramatic enhancement of the A_1g_ stretching intensity signifies strengthened polarizability and altered Cr–O bond covalency, consistent with a Ce-induced redistribution of electronic density within the octahedral structure. Such vibrational strengthening directly influences the magnetic subsystem because the Cr–O–Cr exchange pathways that govern spin ordering are highly sensitive to oxygen displacement and octahedral rigidity. Consequently, the modified octahedral landscape in GCCO enhances the Gd–Cr and Cr–Cr exchange interactions, raising the Néel temperature from 168 K to 173 K and yielding a substantially larger magnetic entropy of − 45.3 JKg^−1^K^− 1^ in the doped sample. Together, these correlated structural and vibrational modifications demonstrate that Ce^3+^ substitution strengthens the coupling between lattice and spin degrees of freedom, thereby amplifying the magnetocaloric response.

**Table 2:** $-\varDelta{S}_{\mathrm{M}}$, RCP of various potential magnetic refrigerant materials having operating temperatures below 20 K along with GdCrO_3_.

Material	µ_0_H (T)	-∆S_M_ (J/kg-K)		T (K)	RCP (J/kg)	Reference
Gd	5	10.2		-	410	^39^
Gd_0.9_Ce_0.1_CrO_3_	5	32.9		3		This work
GdCrO_3_ (single crystal)	4	26.1		5	-	^37^
Gd_0.9_Ce_0.1_CrO_3_	6	37.3		3		This work
La_0.7_Ca_0.3_MnO_3_	2	2.2			55	^40^
La_0.67_Sr_0.1_Ca_0.23_MnO_3_	5	6		-	278.55	^41^
HoCrO_3_	7.2	7		20	408	^42^
DyCrO_3_	4	8.4		15	217	^43^
GdCrO_3_ (polycrystalline)	7	36.9		5	542	^4^
Gd_0.9_Ce_0.1_CrO_3_	7	40.7		3		This work
GdMnO_3_	8	31		19	-	^44^
Gd_0.9_Ce_0.1_CrO_3_	9	45.3		3		This work
HoMnO_3_	7	12.5		10	312	^28^
ErCrO_3_	7	10.7		15	416	^37^
Er_0.33_Gd_0.67_CrO_3_	7	27.6		5	252	^37^
TbCrO_3_	4.5	12.2		4.5	125	^20^
DyCr_0.7_Fe_0.3_O_3_	7	13.1		5	500	^38^
HoFeO_3_ (single crystal)	7	19.2		4.5	220	^45^
HoCr_0.7_Fe_0.3_O_3_	7	6.8		20	387	^38^
Ho_0.67_Tm_0.33_CrO_3_	7	6.7		17	-	^42^
La_0.6_Ca_0.4_MnO_3_	5	8.3		61.2	508	^46^
EuTi_0.9_Cr_0.1_O_3_	2	30		4.2	125	^47^
La_0.7_(Sr, Ba)_0.3_MnO_3_	5	2.8		103.8	285.8	^48^
La_0.57_Nd_0.1_Sr_0.18_Ag_0.15_MnO_3_	5	5.1			146.74	^49^

**Table 3:** Comparative table that highlights the differences in key structural, vibrational, and magnetic parameters of both the GdCrO_3_^31,32^ and GCCO.

Parameter	GdCrO_3_^31^	Gd_0.9_Ce_0.1_CrO_3_
*a*	5.322	5.3194(1)
*b*	5.526	5.5072(1)
*c*	7.623	7.6054(0)
V	223.7(1)	222.8(0)
*out-of-plane bond angle* Cr-O-Cr	146.5˚	145.5˚
*in-plane bond angle* Cr-O-Cr	148.9˚	155.3˚
*t*	0.874	0.866(0)
*s*	0.0373	0.0346(1)
*T*_N_	168 K	173 K
-ΔS_M_ (JKg^−1^K^− 1^)	37.7 (5 K, 7 T)	45.3, 38.7 (3 K, 9 T), (5 K, 7 T)
A_1g_(6) mode CrO_6_ – symmetric stretching	~ 560 cm^− 1^ (*Weak intensity*)^31,32^	~ 557.5 cm^− 1^ (*Highest intensity*)

## Conclusions

In this work, we demonstrate how the typical magnetic landscape of GdCrO_3_ can be manipulated in field, temperature and time domains. Under *H*_DC_, the Cr^3+^ sublattice creates a local field to which the *R*^3+^ sublattice aligns anti-parallel and compensates the magnetization of the Cr^3+^ sublattice which in turn leads to negative magnetization (${M}_{\mathrm{N}}$~ −7.15 emu/g at *T* = 3 K). The overall magnetization undergoes a second anomalous change across the low temperatures associated with spin-flip transition activated by the critical field, *H*_C_ = 200 Oe at ${T}_{\mathrm{S}\mathrm{F}}$= 10 K obtained from the Zeeman interaction term. The distortions brought to reduce the Cr-O-Cr bond angle in the crystal structure by introducing a heavier cation Ce^3+^ at the *A*-site which give rise to an intriguing magneto-caloric effect with change in magnetic entropy as large as 45.3 J/Kg-K (-Δ*S*_M_). Fine tuning of the magnetic landscapes represented by the ${\varGamma}_{4}({G}_{x},{A}_{y},{F}_{z};{F}_{z}^{R})$ and ${\varGamma}_{4}^{{\prime}}$ *w.r.t.* magnetic field and temperature showcase the capability of these candidates in thermo-magnetic switches, magnetic refrigeration, and other spintronic devices.

## Data Availability

The datasets used and/or analyzed during the current study available from the corresponding author on reasonable request.
